# Vehicle-Mounted Vision Sensing for Large-Scale Road Surface Monitoring: A Multi-Year Field Study of Acquisition Geometry and a Heterogeneous Detection–Classification Pipeline for Pothole Mapping

**DOI:** 10.3390/s26175573

**Published:** 2026-09-02

**Authors:** Moon-Sup Lee, Seung-Yeon Han

**Affiliations:** Korea Institute of Civil Engineering and Building Technology, Goyang-si 10223, Republic of Korea; truepath@kict.re.kr

**Keywords:** vehicle-mounted sensing, camera-based sensing, edge AI, computer vision, deep learning, road surface monitoring, pothole detection, heterogeneous cascade, edge inference, intelligent transportation systems

## Abstract

**Highlights:**

**What are the main findings?**

- Acquisition geometry and device cooling strongly affect field pothole-detection yield: an actively cooled windshield mount achieved detection yields of 41.7–58.3%, compared with 0–4.2% for the baseline mount.
- A heterogeneous YOLOv12-large–EfficientNet-B2 cascade reduced the false-positive rate from 99.96% to 32.26%, whereas homogeneous YOLO–YOLO cascades inherit similar biases and fail to suppress common false positives.

**What are the implications of the main findings?**

- In field deployment, camera mounting geometry and thermal management can outweigh incremental detector refinements in determining operational yield.
- Verifying candidate detections with a different-task classifier, rather than with a second detector, suppresses systematic false positives at deployable cost.

**Abstract:**

Vehicle-mounted vision sensing, in which cameras on patrol vehicles acquire ground-level imagery across a road network, offers a low-cost, scalable approach to large-scale road surface monitoring and pothole detection. However, its real-world effectiveness depends not only on detector accuracy on curated imagery but also on two rarely reported factors: field acquisition conditions and pipeline architecture. We present a multi-year field study of a vehicle-based pothole-monitoring system. First, controlled field comparisons show how acquisition geometry and device thermal behavior affect detection yield: across three vehicle types, an actively cooled windshield mount achieved yields of 41.7–58.3%, versus 0–4.2% for a rear-view-mirror baseline. Second, we identify a shared-bias limitation in a homogeneous two-stage detector cascade, in which a second You Only Look Once (YOLO) detector reproduces rather than eliminates first-stage false positives. Third, we redesign the pipeline as a heterogeneous detection-to-classification cascade: an edge-deployed YOLOv12-large detector proposes candidate regions, and a server-side EfficientNet-B2 classifier verifies each. On field data, this raised the detection mean average precision (mAP) from 32.7% to 69.5% and reduced the false-positive rate from 99.96% to 32.26%, cutting manual inspection workload by about 70%. Unlike prior work that mainly optimizes detector architectures, this study shows that acquisition geometry and heterogeneous verification dominate real-world sensing performance under operational deployment.

## 1. Introduction

Potholes threaten road safety and vehicle integrity, and repair costs rise sharply once a defect propagates. Monitoring road-surface conditions across a large network is therefore a large-scale vision sensing problem, and vehicle-mounted cameras have emerged as a low-cost, high-frequency ground-level modality that complements airborne and satellite observation for fine-scale surface defects. Deep-learning-based detection using camera imagery has been widely investigated [1,2,3,4,5], with most studies focusing on detector architectures and performance on curated datasets. In practice, however, a monitoring system deployed on patrol vehicles is constrained by two factors that curated benchmarks do not expose: field-deployment conditions (camera viewing angle, device thermal behavior, vehicle platform, and data-transmission reliability) and the architecture of the detection stack, which must run partly on an edge device and partly on a server.

This paper investigates these two constraints through a multi-year field study that led to the development of a deployable road-monitoring system. We proceed in three stages. Stage one establishes, through controlled field comparisons, that deployment factors dominate real-world yield: Experiment 1 (2022) compared two early single-stage devices against visual ground truth, and Experiment 2 (2024) isolated the camera mount by installing an improved and a baseline mount simultaneously on the same vehicle across three vehicle types. These studies motivated a two-stage design with server-side cross-verification and an improved, actively cooled mount.

Stage two diagnoses a structural limitation that remained after the two-stage design was adopted. In the deployed system, both stages were You Only Look Once (YOLO) object detectors—a lightweight detector on the edge and a larger detector on the server. Because the two stages share architecture, training data, and loss function, they also share their inductive bias: non-pothole objects—such as manholes, shadows, sealed cracks, and tire marks—misclassified by the first stage tend to be confirmed rather than rejected by the second. Enlarging the server model improved localization but did not remove these shared false-positive patterns, so the manual inspection workload remained high.

The third stage addresses this limitation by replacing the homogeneous detector–detector cascade with a heterogeneous detection-to-classification cascade. A high-recall YOLOv12-large detector proposes candidate regions at the edge, and a server-side EfficientNet-B2 binary classifier verifies each candidate as either a pothole or background. Because the verifier is a different task (classification, not detection) trained on a different objective, it does not inherit the detector’s false-positive bias and can therefore suppress the confusions that a second detector could not. We report field measurements of this redesign together with the operational engineering (asynchronous inference decoupling, pre-aggregated reporting, edge-runtime migration) that made it deployable across national road-management offices.

The main contributions of this study are as follows: (1) controlled field measurements quantifying how camera mount geometry and thermal management affect real-world pothole-detection yield across vehicle types; (2) an explicit diagnosis of the shared-bias limitation of homogeneous two-stage detector cascades, grounded in deployed-system observations; (3) a heterogeneous detection-to-classification pipeline (YOLOv12-large + EfficientNet-B2) with field-measured gains in detection mean average precision (mAP) and large reductions in false-positive rate and manual-inspection load; and (4) the supporting edge sensing architecture—Redis-queue asynchronous decoupling, pre-aggregated statistical batching, and a TensorFlow Lite edge-runtime migration—that enables national-scale deployment.

## 2. Related Work

Vehicle-mounted and mobile vision sensing of roads. Monitoring road-surface conditions over a large network is a mobile vision sensing problem, and a broad literature has developed around acquiring geospatial road information from moving platforms. Mobile mapping systems (MMS) that mount laser scanners, cameras, and GNSS/IMU on a survey vehicle are a mature means of collecting road and roadside geometry, and reviews of mobile mapping and surveying technologies document their evolution and accuracy characteristics [6,7]. Beyond dedicated survey vehicles, crowd- and fleet-based sensing uses smartphones and consumer devices—accelerometers, gyroscopes, GNSS, and cameras—to monitor road surfaces at scale, from the early Pothole Patrol mobile sensor network [8] to recent comprehensive reviews of smartphone-based road-surface monitoring [9] and multimodal mobile sensing frameworks [10]. Street-level imagery platforms (e.g., dashcam and panoramic collections) have further enabled large-area, image-based road-surface assessment as a ground-level complement to airborne and satellite observation [11]. Complementary remote-sensing modalities have also been explored for road-surface monitoring, including UAV-based pavement-distress recognition with deep networks [12,13], built-in vehicle cameras integrated with GPS for road-condition mapping [14], and broader reviews of autonomous-vehicle pavement data acquisition and analytics [15]. Our work sits in this vehicle-based remote-sensing tradition but focuses on two under-reported determinants of operational yield—acquisition geometry/thermal behavior and detection-stack architecture—rather than on the sensing platform alone. More recent 2025–2026 studies continue to advance lightweight, edge-deployed road-distress and pothole detection on mobile and vehicle platforms [16,17,18].

Pavement-distress detection. Crack and pothole recognition has been studied with segmentation (e.g., U-Net variants [19]) for thin cracks and with object detection for discrete defects such as potholes, trained on multi-national road-damage datasets [3,4] and smartphone-collected imagery [5], often reviewed comprehensively [20,21]. Recent work spans YOLO-based real-time pothole detectors [22,23], convolutional and transfer-learning classifiers for road damage [24,25,26], and LiDAR- and vision-fusion approaches [27], with comprehensive surveys summarizing the rapid progress [28]. Most reported results emphasize accuracy on curated imagery rather than end-to-end yield under field deployment.

Detectors, cascades, and heterogeneity. Standard architectures include ResNet [29] and Inception [30] classifiers; SSD [31], Faster R-CNN [32], and focal-loss-based dense detectors [33]; transformer-based detectors such as DETR [34] and RT-DETR [35]; and the YOLO family, from the original YOLO [1] to YOLOv7 [2], YOLOv9 [36], YOLOv10 [37], YOLOv11 [38], and YOLOv12 [39]. YOLOv7 and YOLOv12 in particular have demonstrated strong real-time speed–accuracy trade-offs. Two-stage cascades that pair a permissive first stage with a stronger verifier are a well-known means of suppressing false positives. A cascade of two detectors sharing the same architecture, however, tends to share the same inductive bias; pairing a detector with a classifier of a different task is a stronger form of verification. We adopt this heterogeneous detection-to-classification pattern across an edge device and a server.

Deployment factors. Camera viewing geometry, mounting, and thermal throttling of mobile devices are known to affect vision performance, but controlled field measurements of their effect on pothole-detection yield are scarce. This paper contributes such measurements.

## 3. System Architecture

**Figure 1:**
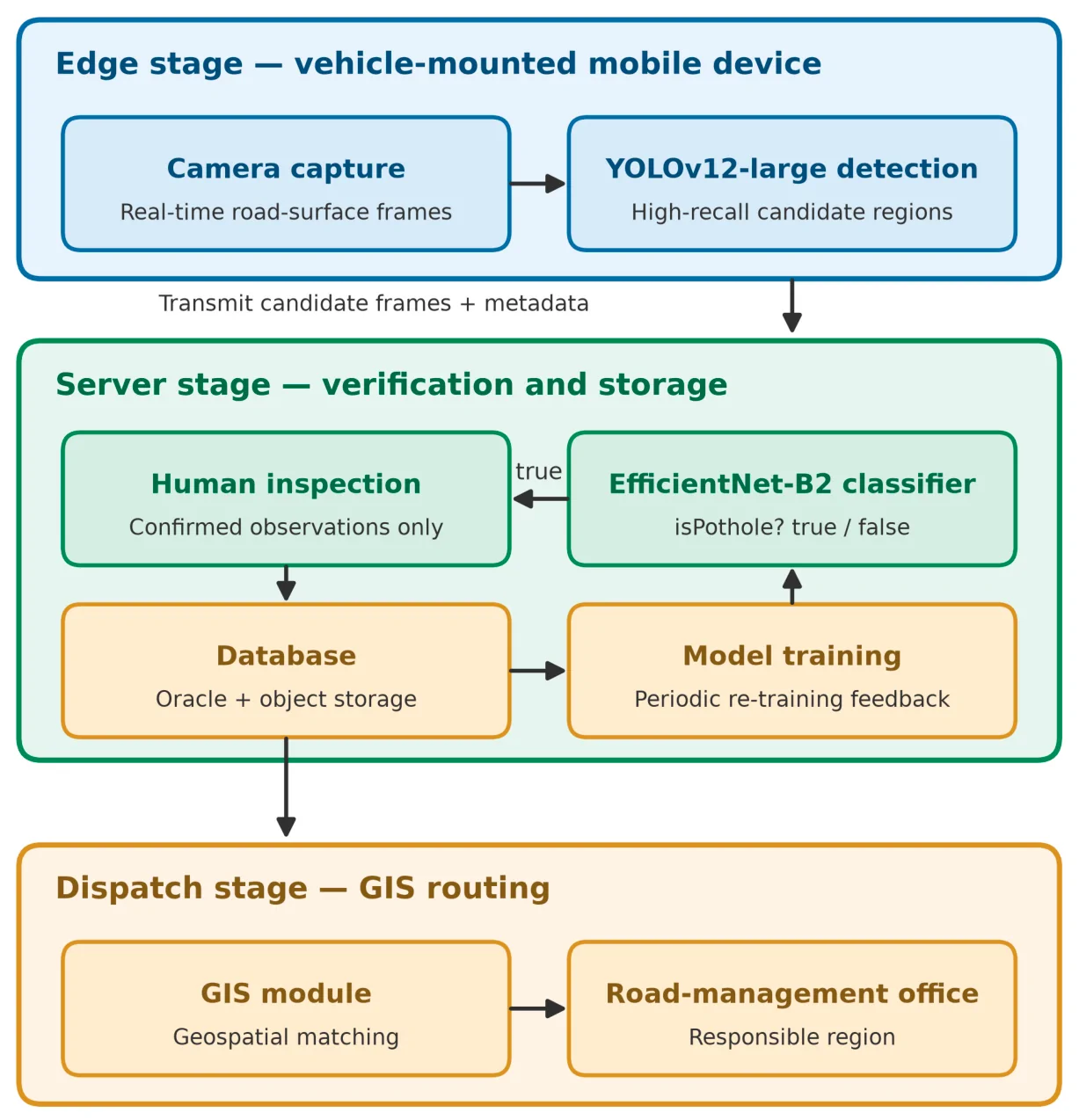
End-to-end sensing architecture, from edge detection to server-side verification and GIS dispatch. Abbreviations: GIS, geographic information system.

**Figure 2:**
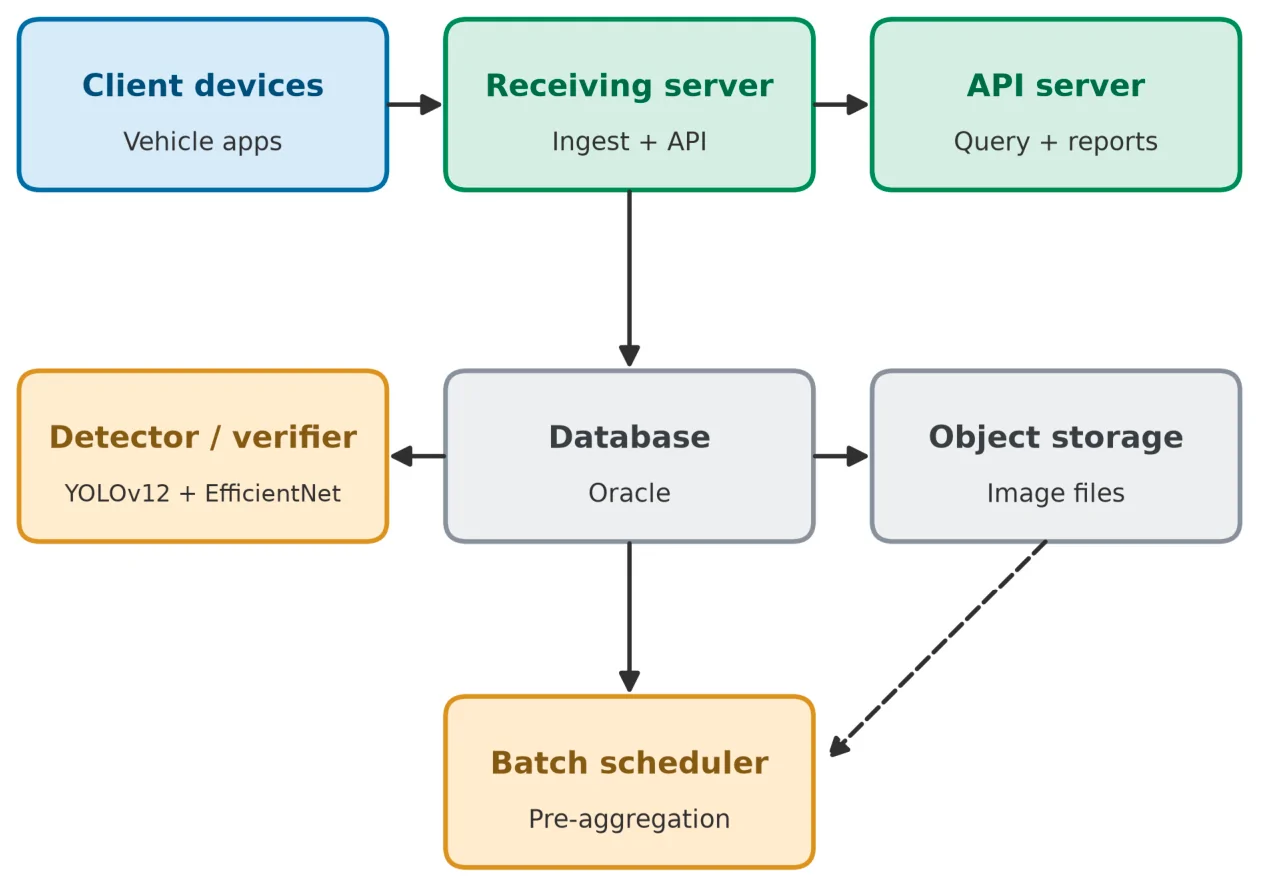
Detection-system architecture and its server-side components.

**Figure 3:**
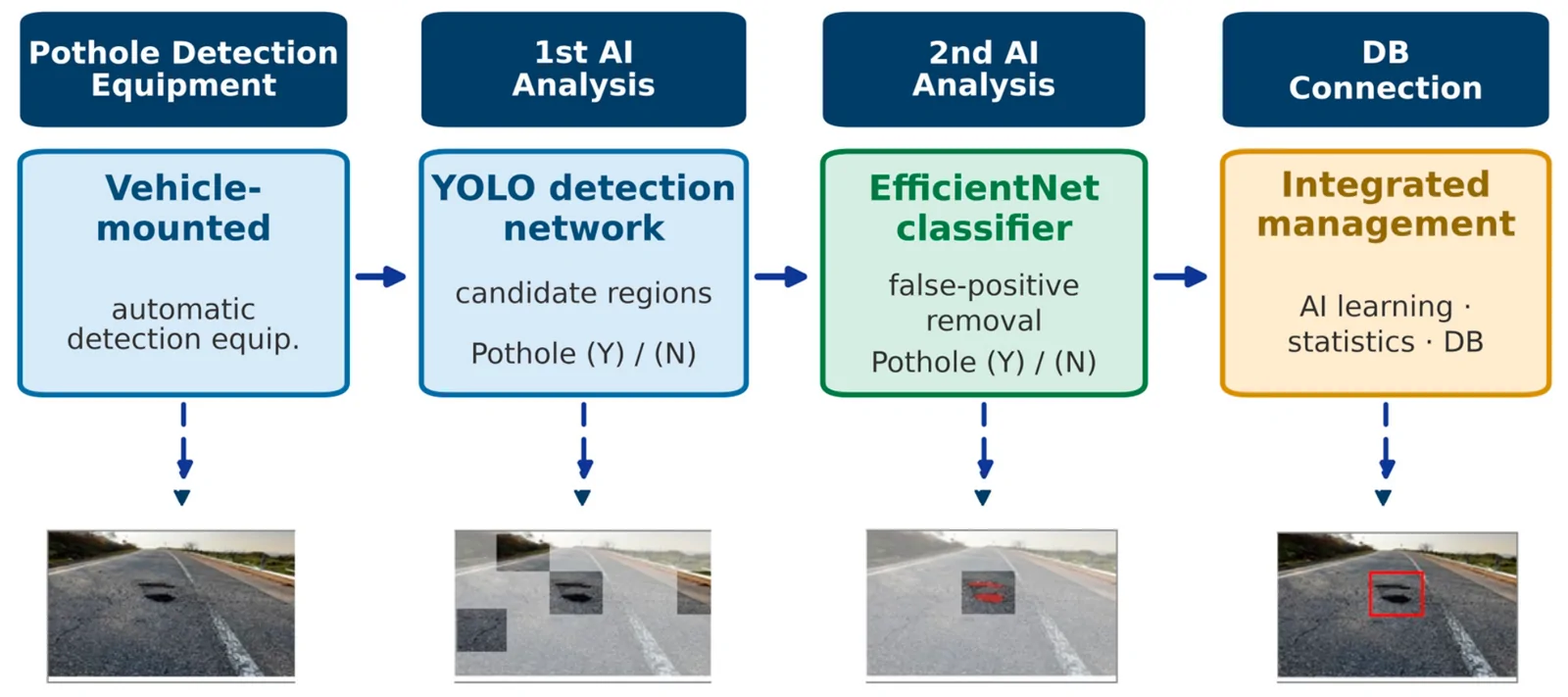
Two-stage verification workflow with independent decisions at each stage. In the final panel, the red box marks the pothole region confirmed by the two-stage pipeline.

The monitoring system automates collection, verification, and regional dispatch of pothole observations from patrol vehicles. A lightweight real-time detector runs on a vehicle-mounted mobile device (edge stage) and transmits candidate frames with metadata; a server stage then verifies each candidate. In the final proposed configuration, the edge detector is YOLOv12-large and the server verifier is an EfficientNet-B2 binary classifier; only observations confirmed by the classifier proceed to human inspection and archival storage (Figure 1, Figure 2 and Figure 3). Confirmed observations are routed by a geographic information system (GIS) module to the responsible road-management office. This architecture is the design response to the experimental findings in Section 5 and to the shared-bias diagnosis in Section 3.2.

### 3.1. Edge and Server Stages

The edge stage performs real-time inference on the vehicle-mounted device and transmits candidate regions (coordinates x, y, w, h) with the associated image path. The server stage re-examines each candidate. Only observations confirmed by the second-stage classifier are archived and dispatched. Table 1 contrasts the deployed homogeneous cascade with the proposed heterogeneous cascade. Persistent storage follows a two-database design with a clear division of roles: an Oracle database holds the detection pipeline’s operational records (candidate frames, classifier decisions, and confidence scores), while a separate PostgreSQL/PostGIS database stores the geospatial layer used for management—confirmed pothole locations, administrative boundaries, and dispatch assignments to the responsible road-management office. Raw and cropped images are kept in object storage and referenced by key from both databases. Confirmed pothole coordinates are matched to the responsible road-management office by a PostGIS spatial join (point-in-polygon against administrative-boundary geometries), and the resulting office assignment is stored with the confirmed observation.

### 3.2. Structural Limit of a Homogeneous Detector Cascade

The system was first deployed as a homogeneous two-stage cascade: a lightweight YOLO detector on the edge and a larger YOLO detector on the server. In operation, this architecture exhibited four limitations. (i) Shared bias: both stages use the same YOLO architecture with similar training data and loss, so the second stage tends to confirm rather than reject the false positives generated by the first stage (manholes, shadows). (ii) Task mismatch: both stages perform object localization, and neither explicitly answers “is this region actually a pothole?”, so a larger model may improve bounding-box localization without improving class discrimination. (iii) Repeated false-positive patterns: manholes, shadows, and sealed cracks are misread as potholes by both stages, and increased model complexity does not translate into false-positive removal. (iv) Cost without commensurate benefit: running a large server detector raises GPU cost and latency while the false-positive rate—and therefore the manual-inspection burden—stays high. These observations motivate replacing the second detector with a different-task verifier.

### 3.3. Proposed Heterogeneous Detection–Classification Cascade

**Figure 4:**
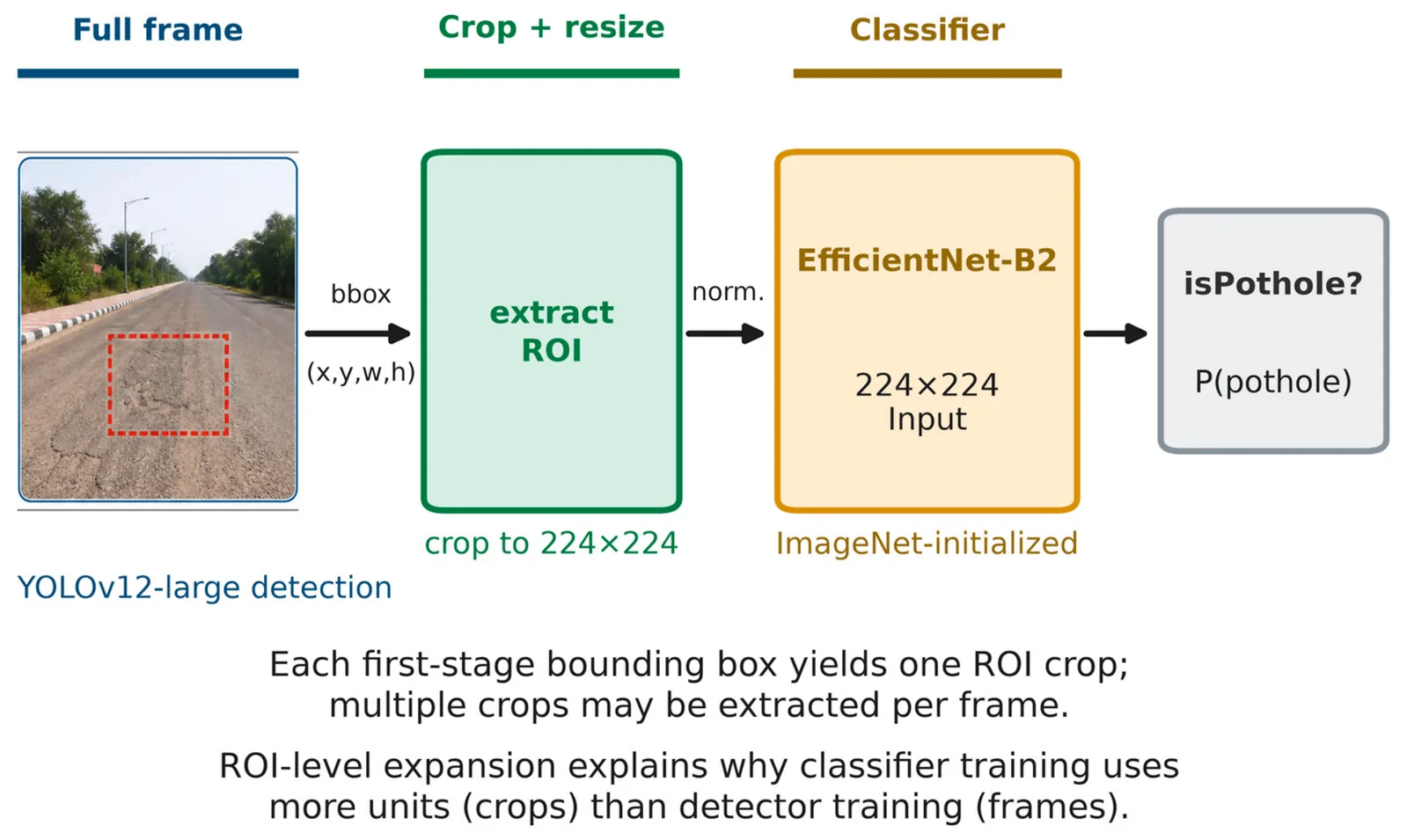
Region-of-interest extraction workflow for second-stage classification, from detector bounding box to 224 × 224 classifier input. The red dashed box in the full frame denotes the first-stage detector bounding box.

The proposed pipeline pairs a high-recall detector with a high-precision classifier. The first stage (YOLOv12-large) generates candidate pothole regions with high recall; the second stage (EfficientNet-B2) classifies each proposed region as pothole or background (Figure 4), filtering out the systematic false positives that a second detector would have confirmed. Because the verifier solves a different task and is trained on a different objective, it does not inherit the detector’s false-positive bias.

The edge detector was upgraded from YOLOv7-tiny [2] to YOLOv12-large [39], whose improved backbone and neck, feature-pyramid [40] changes, and post-processing (non-maximum suppression, NMS [41]) optimizations improve small-object detection while preserving real-time operation. For deployment, the TorchScript model was converted to TensorFlow Lite with mobile optimization and quantization. The server classifier is an EfficientNet-B2 [42] initialized from ImageNet [43] weights and fine-tuned in two stages (feature-extractor stage, then full-network fine-tuning); it is exported to the Open Neural Network Exchange (ONNX) format for inference-time efficiency.

### 3.4. Operational Architecture for Deployment

Asynchronous inference decoupling. In the original design, the server called the second-stage AI synchronously and waited for its response, so any AI delay or fault propagated into the request path. The pipeline was re-architected around a Redis queue: the server persists first-stage data and enqueues a request, a separate AI worker consumes the queue and performs second-stage verification, and a result consumer writes the outcome back to the database. Request, result, and error queues are separated, so AI faults are isolated from the request path, failed messages are retained for retry, and the number of workers can be scaled horizontally.

Pre-aggregated statistical batching. Report queries over a table holding more than two million JSON location records caused full-table scans and disk-I/O bottlenecks. Statistics were migrated from the operational tables to a pre-aggregated store updated through daily batch processing, ensuring that report latency remains approximately constant as the dataset grows and no longer competes with the data-ingestion workload.

Edge-runtime migration. To satisfy updated store policies (target API level, 16 KB memory-page support) and to remove a runtime that could not be rebuilt for 16 KB pages, the on-device inference stack was migrated from PyTorch Mobile to TensorFlow Lite (LiteRT) [44], with selective CPU/NPU acceleration; sensitive tokens were moved to encrypted on-device storage, and crash/usage telemetry was added for visibility into field failures.

## 4. Materials and Methods

### 4.1. Overview of the Field Studies

Three sources of field evidence were used. Experiment 1 (May 2022) compared two early single-stage detection devices—an action-camera-plus-laptop system and a compact IoT device—against visual ground truth. Experiment 2 (May 2024) compared an improved camera mount against a baseline mount, installed simultaneously on the same vehicle and repeated across three vehicle types. The proposed heterogeneous pipeline (Section 3.3) was then evaluated using operational field data collected in 2025. Experiments 1 and 2 establish the deployment-factor and cross-verification rationale; the 2025 evaluation establishes the performance of the final proposed system. Visual road inspection was used as the ground truth in all studies.

### 4.2. Study Site and Protocol (Experiment 2)

**Figure 5:**
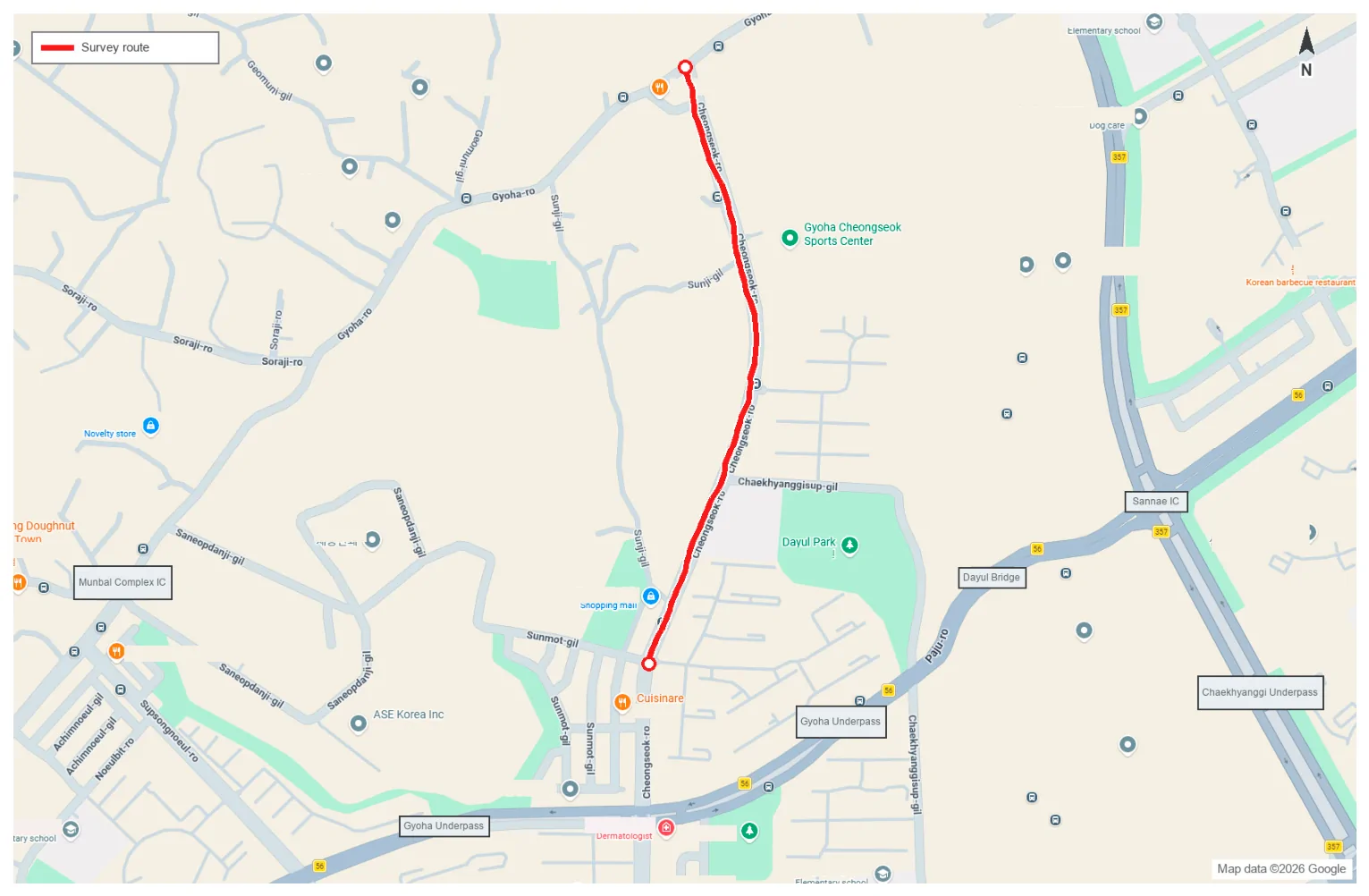
Experiment 2 study route: a 2 km out-and-back segment in Dayul-dong, Paju (traversed three times per vehicle).

Experiment 2 was conducted on a fixed 2 km out-and-back route in Dayul-dong, Paju, Gyeonggi-do (Figure 5). Each vehicle traversed the route three times (total 6 km). Ground truth comprised 8 unfilled potholes and 22 repaired potholes along the route; with three passes, this resulted in 24 and 66 pothole encounters, respectively, which served as the denominators for the yield calculations. Three vehicle platforms were tested—a truck (Porter; Hyundai Motor Company, Seoul, Republic of Korea), a van (Starex; Hyundai Motor Company, Seoul, Republic of Korea), and a passenger car (K3; Kia Corporation, Seoul, Republic of Korea)—to represent different camera heights and vibration characteristics.

**Figure 6:**
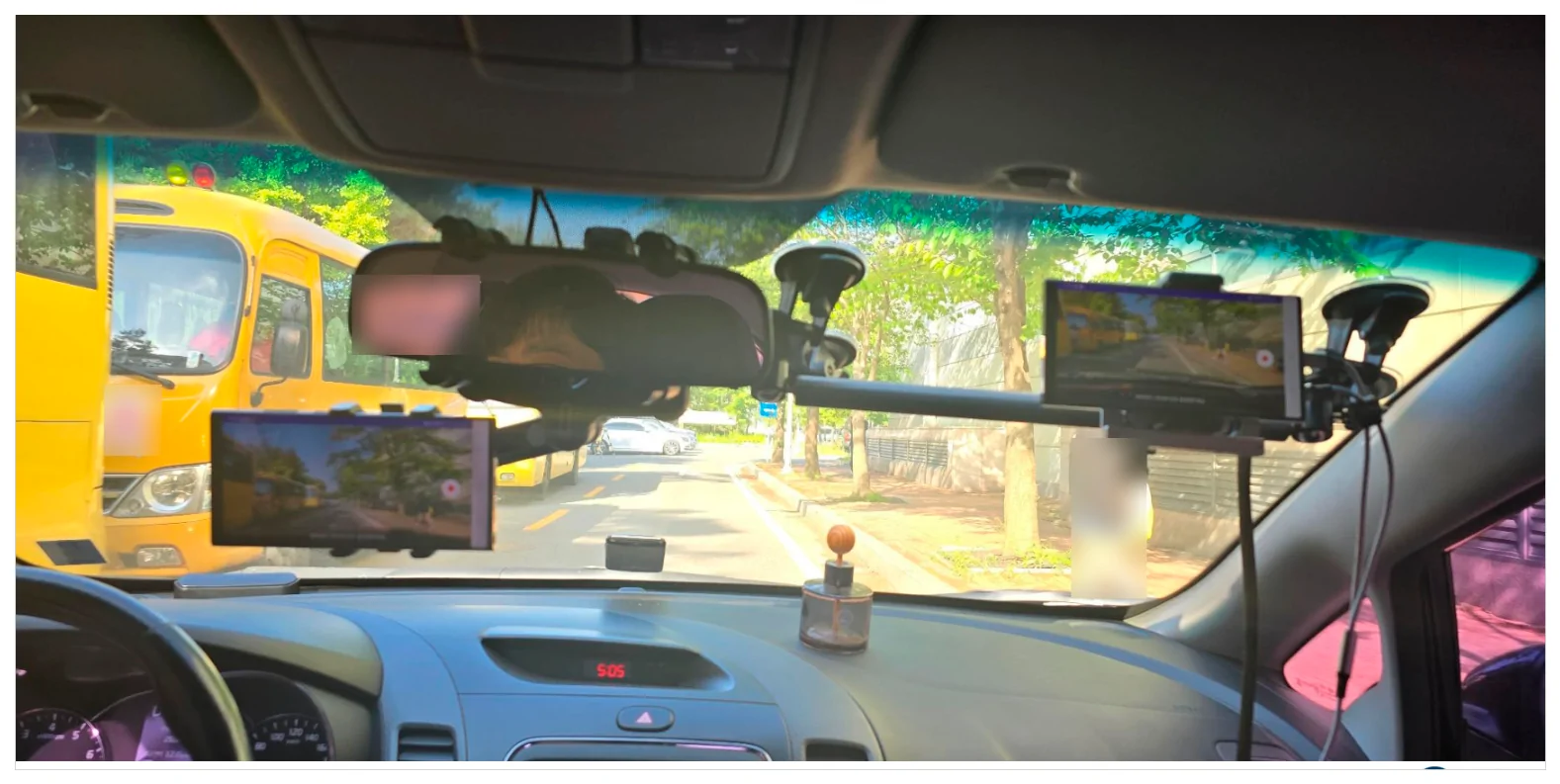
Simultaneous installation of the baseline (rear-view-mirror) and improved (passenger-side windshield position with four-fan forced-air cooling) mounts on the same vehicle, ensuring identical conditions.

On each vehicle, the baseline mount (rear-view-mirror position, wireless charging, and a cooling plate) and the improved mount (passenger-side windshield position, four-fan forced-air cooling) were installed and operated simultaneously so that both experienced identical road, lighting, and traffic conditions (Figure 6). Detected frames transmitted to the server were compared against the visually confirmed potholes.

### 4.3. Evaluation Metric: System-Level Detection Yield

For Experiment 2, we report a system-level detection yield, defined as the number of ground-truth potholes correctly detected and received at the server, divided by the total number of ground-truth pothole encounters (potholes times passes). Encounters for which no data reached the server—whether due to device malfunction, thermal shutdown, or transmission failure—are counted as misses (they contribute to the denominator but not the numerator). This metric measures end-to-end operational effectiveness of the deployed pipeline rather than isolated detector accuracy. It conflates detection failure with non-reception; the implications of this definition are discussed in Section 6. For transparency, Table 2 additionally reports raw detected and missed counts, where missed = encounters − detected and therefore aggregates both model non-detection and non-reception. Because the ground-truth sample is small, each yield is accompanied by a 95% bootstrap percentile confidence interval [45] (20,000 resamples of the per-encounter Bernoulli outcomes, with a fixed random seed for reproducibility).

### 4.4. Detectors, Verifier, Training, and Datasets

**Figure 7:**
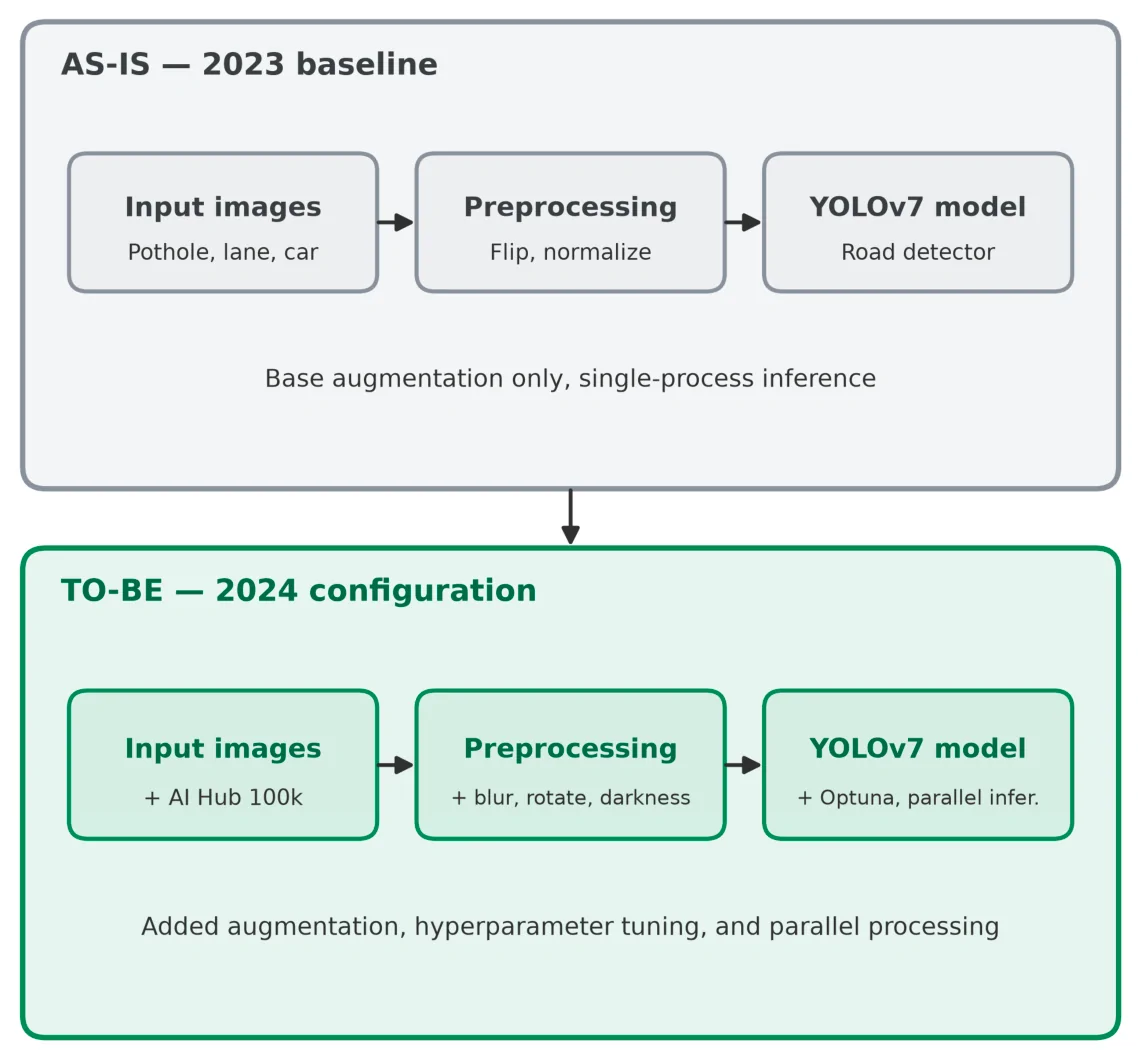
Detector-training process improvement from the 2023 baseline to the 2024 configuration.

Deployed cascade (background). The originally deployed server detector was YOLOv7 [2], selected after comparison with alternatives based on ResNet [29], Inception v3 [30], and SSD [31] for its real-time speed–accuracy trade-off. Base augmentation comprised vertical/horizontal flips and normalization; an extended configuration added blur, rotation, and low-light augmentation, expanded training with approximately one hundred thousand public-road-obstacle/surface images (AI Hub [46]; complemented by public multi-national road-damage datasets such as RDD2022 [3]), applied automated hyperparameter tuning (Optuna [47]), and parallelized inference to reduce thermal load (Figure 7). A post-processing step excluded painted lane markings and construction joints via a grayscale intensity band (195–255) with shape recognition and a Hough-based straightness test.

Proposed pipeline. The first stage is YOLOv12-large, trained on 15,609 frames (2856 existing plus 12,753 newly collected operational frames). The second stage is an EfficientNet-B2 binary classifier operating on 224 × 224 region-of-interest (ROI) crops, initialized from ImageNet weights and fine-tuned in two stages; it outputs probabilities for the background and pothole classes. The classifier training set comprises 58,031 ROI crops (8564 background crops, including manholes, shadows, cracks, tire marks; 49,467 pothole crops) with a 15,112-crop validation set. The first-stage and second-stage counts are expressed in different units—whole frames for detection training versus cropped candidate regions for classification training—so the larger classifier count reflects multiple ROI crops extracted per frame rather than a larger image collection. Full dataset, augmentation, cropping, export, and training details are given in the Supplementary Materials (Supplementary Text S1–S9).

### 4.5. Model Selection Rationale and Alternatives

First stage (detector). The edge stage must run in real time on a vehicle-mounted device while retaining enough recall that few true potholes are lost before verification. Among real-time single-stage detectors, the YOLO family offers the best speed–accuracy operating point for this constraint. We upgraded from YOLOv7-tiny to YOLOv12-large because the newer backbone and neck, improved feature-pyramid design, and post-processing (NMS) optimizations raise small-object recall—the dominant field failure mode identified in Section 5.3—while remaining exportable to a quantized TensorFlow Lite model for on-device inference. A larger two-stage detector such as Faster R-CNN [32] was not adopted for the edge because its latency is incompatible with real-time capture; SSD [31] and older YOLO variants [1] were rejected for weaker small-object recall at comparable speed. Intermediate YOLO variants (e.g., the programmable gradient information of v9 and the NMS-free design of v10) were not adopted because, in our edge-export tests, they did not offer a comparable recall-per-latency gain under the quantized TensorFlow Lite export required by the target on-device deployment; the stable quantized export path was a decisive practical factor alongside small-object recall.

Second stage (verifier). The central design decision is that the verifier is a classifier, not a detector. As diagnosed in Section 3.2, a second detector sharing the first stage’s architecture and loss inherits its false-positive bias and therefore confirms rather than removes shared confusions (manholes, shadows, sealed cracks). A binary classifier trained on a different objective breaks this shared bias. Within classifiers, EfficientNet-B2 was selected as a practical compromise between classification accuracy, model complexity, and inference latency: its compound-scaled architecture provides strong ImageNet-pretrained performance while remaining compatible with the parameter and latency constraints of the queue-based server environment, and it exports cleanly to ONNX. Lighter backbones (e.g., MobileNet-class [48,49]) were considered for lower latency but offered weaker fine-grained discrimination between potholes and visually similar road-surface features; heavier backbones (e.g., EfficientNet-B4/B5 [42,50] or large ResNets [29]) improved capacity marginally but raised latency without a commensurate false-positive reduction in preliminary checks, and capacity was not the binding constraint—task diversity was.

The choice of EfficientNet-B2 as the second-stage verifier was based on three qualitative considerations rather than a single accuracy figure. First, the verifier must add task heterogeneity: because the first stage is a YOLO detector, a classifier trained on a different objective (whole-region background-versus-pothole classification, not bounding-box regression) contributes an independent decision boundary and does not merely reproduce the detector’s inductive bias. Second, the operating point must fit an asynchronous server behind a queue, where the primary constraints are stable throughput and a favorable accuracy-per-parameter trade-off rather than the lowest possible latency; EfficientNet’s compound scaling places the B2 variant at a balanced point on this curve, heavier than mobile-class backbones yet far smaller than large ResNets. Third, fine-grained texture discrimination is decisive for this task, since the dominant false positives (manhole covers, shadows, tar seams, lane markings) are separated from potholes by subtle surface-texture cues; the feature hierarchy of EfficientNet-B2 captured these distinctions more reliably than lighter mobile-class backbones in preliminary qualitative review, while the incremental capacity of B4/B5 did not change the decisions that mattered. A controlled head-to-head benchmark of alternative verifiers on a common region-of-interest set has now been carried out on the recovered region-of-interest dataset and is reported in Table 3; the measured collapse of the second-stage false-positive rate reported in Section 5.4 and summarized in Table 4 provides complementary field evidence. Table 3 reports a matched-ROI comparison of five candidate verifier backbones—MobileNetV3-Large, ResNet-50, and EfficientNet-B2/B4/B5—trained and evaluated on the recovered region-of-interest dataset (58,031 training/15,112 validation crops) under a single identical protocol (input 224 × 224 with ImageNet normalization matching the deployed verifier, class-weighted cross-entropy for the 5.8:1 background-to-pothole imbalance, AdamW at a 1 × 10^−4^ learning rate, batch size 128, and six epochs). The candidate backbones perform comparably, with pothole-class F1 between 86.7% and 90.5%: the deployed EfficientNet-B2 attains an F1 of 89.3% (precision 92.7%, recall 86.1%) at 7.7 M parameters, the lightweight MobileNetV3-Large is competitive (F1 90.5%, 4.2 M), and the larger ResNet-50 and EfficientNet-B4/B5 provide no F1 improvement despite two to four times the parameters. EfficientNet-B2 therefore occupies a strong accuracy-per-parameter operating point, and increasing backbone capacity yields no accuracy benefit on this ROI-verification task.

The alternatives in Table 5 were assessed qualitatively from the design rationale of the deployment program; the decisive quantitative evidence is the change in behavior when each stage was replaced under matched operating conditions, summarized as a component-level ablation in Table 4. The ablation isolates two changes measured on field data: upgrading the first-stage detector (YOLOv7-tiny → YOLOv12-large) and, independently, changing the second-stage verifier from a YOLOv7 detector to an EfficientNet-B2 classifier. The first change raises first-stage detection quality (mAP, recall, precision); the second substantially reduces the false-positive rate, which is the quantity a same-task second detector could not reduce. Taken together, these results indicate that the accuracy gain and the false-positive reduction come from different components, and that the false-positive reduction is attributable specifically to introducing task heterogeneity at the verifier rather than to added detector capacity.

### 4.6. Implementation Environment

Development and inference used an Intel Core i9-10900X (10-core, 3.7 GHz; Intel Corporation, Santa Clara, CA, USA), 64 GB DDR4 memory, an NVIDIA GeForce RTX 3060 (12 GB; NVIDIA Corporation, Santa Clara, CA, USA), and a 1 TB SSD. The deployed-cascade software stack used CUDA 10.1, cuDNN 7.6.4, a Qt 5+ application layer, Python 3.7+, PyTorch 1.7.1, TensorFlow 2.2.0, and segmentation-models-pytorch 0.2.1. The proposed edge runtime uses TensorFlow Lite (LiteRT) with quantization; the server verifier is exported to ONNX. The exact model of the server GPU and the specific vehicle-mounted edge device (make, model, and system-on-chip) are not documented in the retained project records and are therefore left unspecified here rather than estimated. On-target inference latency was not profiled because the deployed trained weights and the field hardware are not part of the recovered materials (Section 6); we therefore report the deployment configuration—a quantized TensorFlow Lite edge detector and an ONNX server verifier with asynchronous Redis-queue decoupling (Section 3.4)—and identify rigorous on-target latency benchmarking (edge ms/frame and FPS, server ms/crop, and end-to-end latency, including transmission) as future work.

### 4.7. Evaluation Protocol and the Distinct Measurement Populations

This study reports measurements drawn from several distinct populations collected over multiple years, and they are not directly comparable. To prevent misreading, Table 6 states, for each reported result, the population, sample size, year, and ground truth. Two consequences follow. First, the system-level yield of Experiment 2 (a route-level operational metric on the 2024 Paju route) and the model-level accuracy metrics (mAP, precision, recall on curated image sets) answer different questions and must not be compared numerically. Second, within the model-level track, the first-stage detection test (276 images) and the second-stage false-positive rate (operational driving data collected in 2025) use different frames and different denominators, so the first- and second-stage numbers describe complementary aspects of the pipeline rather than a single end-to-end score. A controlled unified held-out end-to-end evaluation of the recovered cascade is now reported in Section 5.6 (Table 7) on 2550 unseen frames; the historical measurements reported elsewhere in this manuscript nevertheless arise from distinct populations and should not be compared numerically across years or denominators.

## 5. Results

### 5.1. Experiment 1: Comparison of Early Single-Stage Devices (2022)

**Figure 8:**
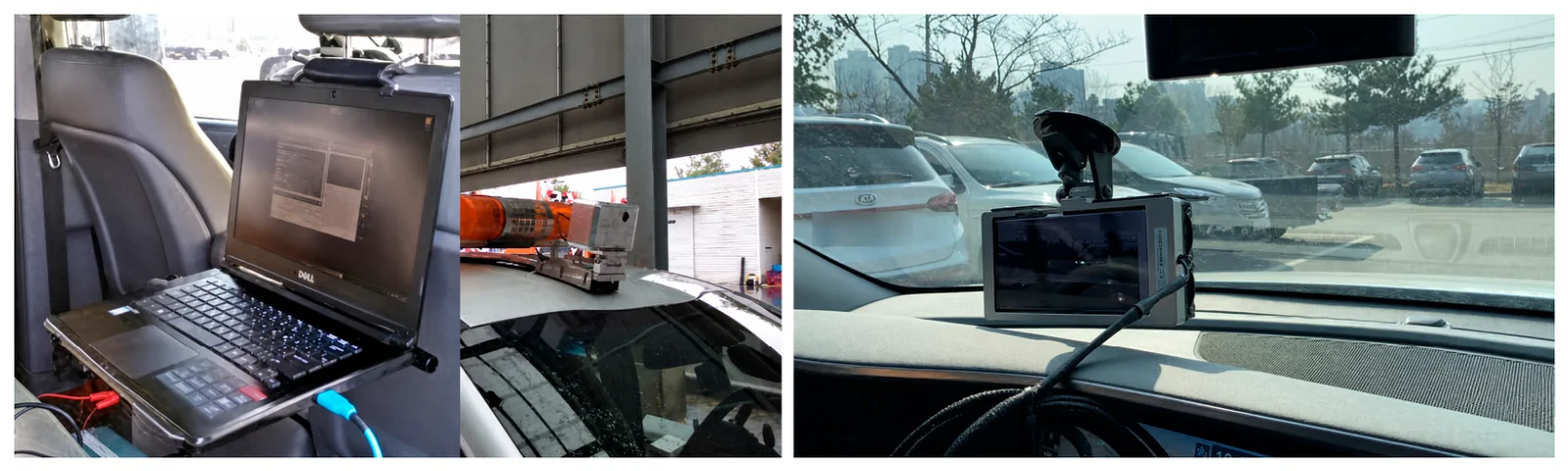
Early detection devices compared in Experiment 1: action-camera-plus-laptop system (**left**) and compact IoT device (**right**).

Two early single-stage devices (Figure 8) were evaluated against visual ground truth on a common route (Table 8). The action-camera/laptop device transmitted 32 predicted potholes, of which only 7 (22.0%) were verified as real, implying a 78% false-positive rate. The compact IoT device transmitted 370 predicted detections, but KICT verification confirmed 0 (0.0%) as real potholes under both automated and manual review. Both single-stage devices thus exhibited either very high false-positive rates or near-zero verified yield, directly motivating a two-stage design in which a second, server-side stage verifies first-stage candidates before they are accepted. The contrast between the two devices is statistically significant: a two-sided Fisher’s exact test comparing the verified and unverified counts for the action-camera system (7/32) and the compact IoT device (0/370) yielded *p* < 0.001, indicating a statistically significant difference in verified yield between the two devices.

### 5.2. Experiment 2: Vehicle-Type × Mount Controlled Yield (2024)

With the improved and baseline mounts installed simultaneously on the same vehicle, system-level detection yield (Section 4.3, non-reception counted as a miss) was measured for three vehicle types (Table 2). For every vehicle type and both pothole conditions, the improved mount substantially outperformed the baseline. For unfilled (pre-repair) potholes, the improved mount achieved 41.7–58.3% yield versus 0–4.2% for the baseline; for repaired potholes, 19.7–30.3% versus 0–7.6%. Because the two mounts operated under identical road, lighting, and traffic conditions on the same vehicle, the observed difference is most plausibly associated with the combined effects of mounting position and cooling configuration—the improved mounting position and forced-air cooling, which reduces thermal throttling and non-reception during continuous operation.

Interpretation. The baseline mount yielded near-zero pre-repair detections for two of three vehicle types, largely because little or no data were received during those runs; the improved mount both widened the forward field of view and, through active cooling, sustained transmission over the full route. In this experiment, the combined mounting and thermal-management configuration had a larger effect on system-level yield than the incremental detector improvements examined separately.

Uncertainty of the yield estimates. Because the ground-truth sample is small, each yield is reported with a 95% bootstrap percentile confidence interval (20,000 resamples of the per-encounter Bernoulli outcomes; Table 9). We treat these intervals as exploratory rather than confirmatory: they characterize the sampling uncertainty of each cell, not a powered hypothesis test. Two observations are robust to this uncertainty. First, for every vehicle type and both pothole conditions, the improved-minus-baseline yield difference has a 95% interval that lies entirely above zero (the smallest, truck-repaired, is +12.1 percentage points, (95% confidence interval (CI) [1.5, 24.2]), so the direction of the mount effect is consistent across all six comparisons. Second, pooling the three vehicles for pre-repair potholes gives an improved yield of 51.4% (95% CI [40.3, 62.5]) versus a baseline of 1.4% (95% CI [0.0, 4.2]); the intervals do not overlap. For cells with zero observed detections (e.g., truck and passenger-car baseline, pre-repair), the percentile bootstrap degenerates to [0.0, 0.0] because every resample of an all-zero sample is zero; these are reported as observed zeros rather than as genuinely tight intervals, and a rule-of-three upper bound (~12.5% for *n* = 24) is a more appropriate upper bound on the detection probability that can be excluded by the available sample.

### 5.3. Failure Analysis

**Figure 9:**
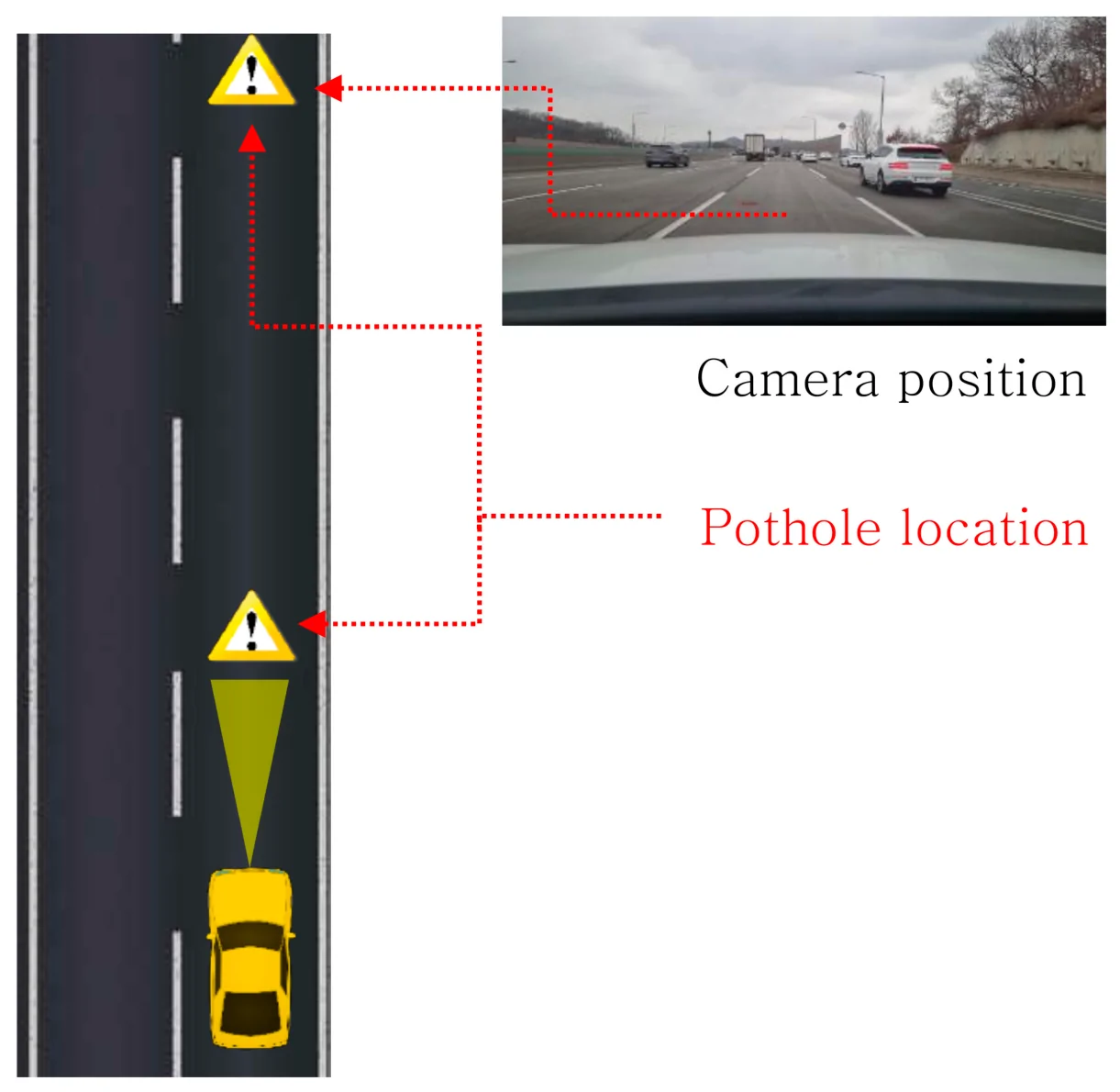
Failure mode 1: a small pothole below the size threshold is missed; capture position affects apparent size, motivating distance-aware size learning.

**Figure 10:**
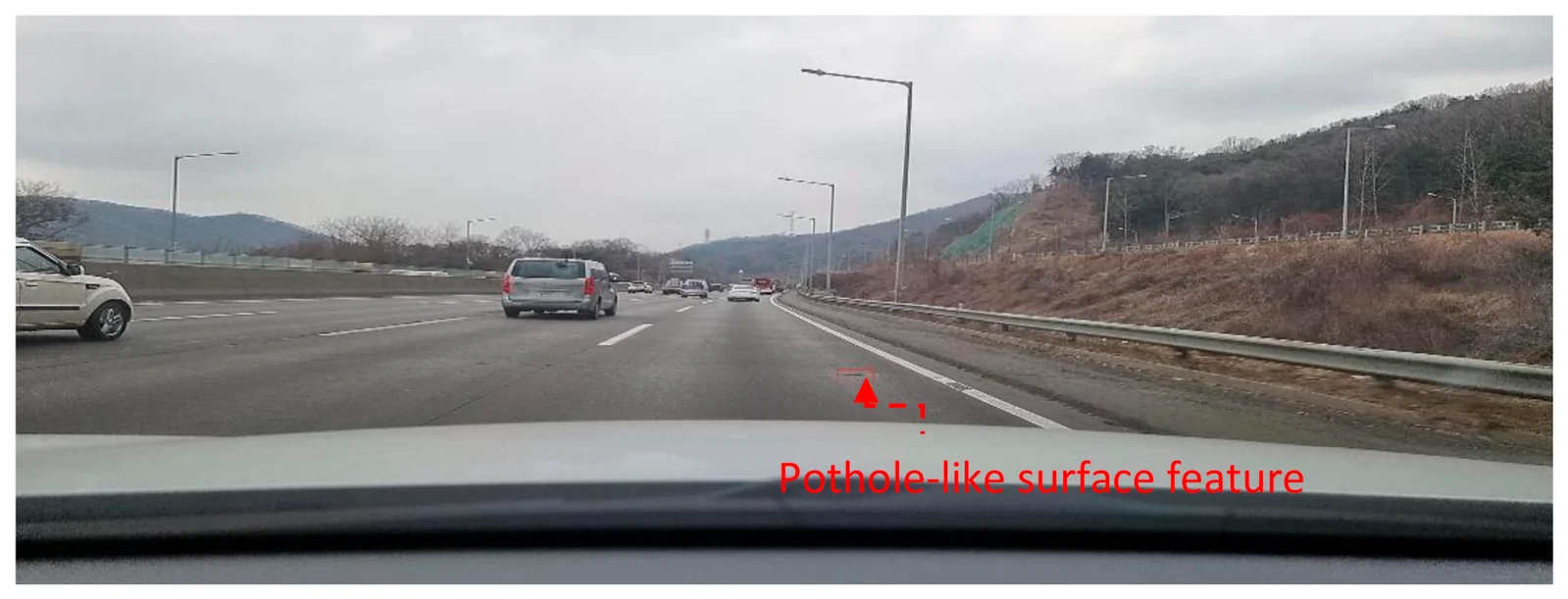
Failure mode 2: a similar-appearance object detected as a candidate but visually confirmed not to be a current pothole; retained for separate tracking.

Two dominant failure modes were identified. First, small potholes whose apparent size fell below the detector’s size threshold were missed, especially at greater distances; this motivates position-dependent (distance-aware) size learning so that small, far potholes are retained (Figure 9). Second, the system sometimes detected similar-appearance objects that visual inspection confirmed were not current potholes but exhibited surface characteristics considered relevant for continued monitoring; rather than discarding these, the pipeline tracks them as a separate category for monitoring (Figure 10). These observations motivate the three-valued inspection label (present/absent/repaired) and tracking of pothole-like objects described in the architecture, and—together with Experiment 1—they motivate the classification-based verifier that directly answers “is this region a pothole?” We note that the verifier itself is a binary classifier (pothole versus background); the “pothole-like” objects are not a third classifier class but are handled by separate operational logic—the three-valued inspection label (present/absent/repaired) and a monitoring flag applied at the review and archival stage.

### 5.4. Proposed Heterogeneous Pipeline: Field Performance (2025)

The proposed detection-to-classification pipeline was evaluated using operational field data collected in 2025. Upgrading the first stage from YOLOv7-tiny to YOLOv12-large raised detection performance substantially on a 276-image test set using a confidence threshold of 0.1 and an intersection-over-union (IoU) threshold of 0.4 (Table 10). Replacing the second-stage YOLOv7 detector with an EfficientNet-B2 classifier reduced the second-stage false-positive rate from 99.96% to 32.26% on operational driving data collected in 2025—a 67.7-percentage-point reduction—while the classifier itself achieved 73.6% precision, 74.3% recall, and an F1-score of 73.9% (Table 11). For the classifier, we report precision, recall, and F1-score rather than mAP, since mean average precision is a detection metric and is not well defined for a binary region classifier. In operation, the redesign reduced the volume of detections requiring manual inspection by roughly 70%, directly addressing the shared-bias limitation diagnosed in Section 3.2.

### 5.5. Two-Stage Detection Metrics of the Deployed Cascade (Background)

For completeness, Table 12 reports detection metrics of the earlier deployed cascade that preceded the redesign. First-stage (app) and second-stage (server filter) detection each reached mAP near 0.57–0.60, and raw detection count fell from 186 to 122 as precision-oriented filtering was applied—consistent with progressive false-positive suppression, and corroborating the Experiment 1 finding that false positives, not misses, were the principal early-stage problem. These figures belong to the homogeneous cascade and are superseded by the proposed pipeline in Section 5.4.

### 5.6. Controlled Held-Out End-to-End Evaluation of the Cascade

To complement the operational false-positive measurement of Section 5.4 with a controlled, unified comparison, we evaluated the full cascade end-to-end on a held-out test set of 2550 recovered frames that were used in no training. The first-stage detector proposes candidates, each candidate region of interest is verified by the EfficientNet-B2 classifier, and surviving detections are matched to ground truth at an intersection-over-union of 0.5. For this reproduction, the detector and verifier were retrained on the training split alone, so the held-out frames are unseen by both stages. As reported in Table 7, adding the heterogeneous verifier reduced false positives by 27.3% (2745 to 1996) and raised precision from 0.709 to 0.755 while retaining 91.7% of detector recall (0.663 to 0.608). Because the second stage can only remove candidates, it acts as a precision-oriented filter by design, trading a small amount of recall for a substantial reduction in false positives—consistent with the operational objective of minimizing false inspection dispatches. These matched held-out figures are a controlled reproduction on the recovered data and use a per-detection metric that is distinct from, and complementary to, the operational second-stage false-positive rate reported in Section 5.4.

## 6. Discussion

Deployment factors dominate yield. The clearest field result is that the camera mount—its forward angle and thermal management—was more influential in the controlled field comparison than the incremental performance differences observed among detector configurations, consistently across three vehicle platforms (Table 2). This reframes “improving pothole detection” for fielded systems: gains available from mounting and thermal design can exceed those from model tuning, yet are rarely reported.

Heterogeneity, not size, removes false positives. Enlarging the server detector within a homogeneous cascade improved localization but not false-positive removal because both stages shared the same bias (Section 3.2). Replacing the second detector with a different-task classifier cut the second-stage false-positive rate and reduced manual-inspection load by roughly 70% (Section 5.4). These findings suggest that for verification, task diversity matters more than model capacity.

Why two stages at all. Experiment 1 showed early single-stage devices failing in opposite ways—one with 78% false positives, the other with near-zero verified yield. A cascade that pairs a permissive edge detector with a stronger verifier addresses both; the heterogeneous verifier addresses the false-positive failure mode more effectively than a second detector could.

Failure modes and pipeline response. Size-threshold misses and similar-appearance false objects (Section 5.3) motivate distance-aware size learning and a three-valued inspection with tracking of pothole-like objects, tying the observed errors directly to concrete pipeline mechanisms.

Operational engineering enables deployment. Asynchronous Redis-queue decoupling isolates AI faults from the request path and allows workers to scale; pre-aggregated batching keeps report latency roughly constant as data grow; and the TensorFlow Lite edge migration keeps the app deployable under changing store policies. These operational improvements are essential for practical deployment because they make model-level accuracy gains usable in national-scale operation.

Transferability to mobile vision sensing beyond this network. Although the field measurements come from one national road network, the findings may be relevant to vehicle-mounted mobile vision sensing more broadly. Acquisition geometry (viewing angle, platform height, vibration) and sensor thermal stability are platform-level factors that condition ground-level image quality irrespective of the specific road or detector, so the observation that they dominate operational yield is a transferable lesson rather than a local artifact. Likewise, the shared-bias limitation of a homogeneous cascade and its resolution by task-heterogeneous verification are properties of the modeling stack, not of the study site, and apply to any mobile sensing task where a permissive detector must be verified. We therefore frame the system-level yield metric, the acquisition-geometry effect, and the detection-to-classification design as reusable guidance for large-area ground-level vision sensing of road-surface condition. Concretely, the acquisition-geometry and thermal-stability findings transfer to UAV-based pavement inspection (viewing angle, airframe vibration, and on-board thermal throttling) and to tunnel-surface monitoring (low-light standoff imaging under continuous operation), while the task-heterogeneous detection-to-classification verification generalizes to other mobile visual sensing pipelines in which a permissive detector must be verified—subject to domain-shift caveats (illumination, standoff distance, and motion characteristics) that would require per-platform re-calibration. These examples are illustrative rather than experimentally validated applications.

Limitations. (i) The Experiment 2 yield metric counts non-reception as a miss and therefore conflates detection failure with device/transmission failure; the baseline mount’s near-zero pre-repair yields reflect, in part, unreceived data rather than pure model error, and the two failure types should be separately instrumented in future work. (ii) Sample sizes for the controlled comparisons are small (Experiment 1: tens of candidates; Experiment 2: 8 and 22 ground-truth potholes over three passes). We therefore report bootstrap confidence intervals (Table 9) and treat the comparisons as exploratory field rather than statistically powered; although the improved-minus-baseline direction is consistent across all six comparisons with intervals above zero, a larger multi-route trial is needed for confirmatory inference, and the zero-detection cells in particular cannot be tightly bounded from the present sample. (iii) The evaluation populations are not uniform across measurements: Experiment 2 yield is measured on the Paju route (2024); the first-stage detector figures on a 276-image test set; the second-stage false-positive rate on operational driving data collected in 2025; and the deployed-cascade metrics on a 100-image set. These differ in year, size, and sampling frame, so cross-comparison must be qualitative. Table 6 makes the correspondence explicit, and Section 5.6 additionally reports a controlled unified held-out end-to-end evaluation of the recovered cascade on unseen frames; the historical operational and model-level measurements collected between 2022 and 2025 nevertheless differ in year, size, and sampling frame, so cross-comparison among them remains qualitative. (iv) The two-database design (Oracle for operational detection records, PostgreSQL/PostGIS for the geospatial management layer) is an intentional separation of the real-time detection store from the spatial management store, described in Section 3.1. Finally, although the present study focuses on pothole detection, the proposed deployment framework and heterogeneous verification strategy are applicable to other vehicle-based remote-sensing tasks requiring large-scale visual infrastructure monitoring. (v) The region-of-interest dataset and the deployment pipeline were recovered from the development contractor, which enabled the matched-ROI backbone comparison now reported in Section 4.5 and direct documentation of the classifier preprocessing. A few items still depend on data not present in the recovered materials—the exact numeric hyperparameters of the original production training, on-target edge and server inference latency (which requires the deployed trained weights and field hardware), and per-encounter operational telemetry for disaggregating hardware from model error—and these are marked as future work; all configuration details retained in the project records are reported.

## 7. Conclusions

This multi-year field study demonstrated that camera mounting geometry and thermal management strongly influence the operational yield of a vehicle-based pothole-monitoring system. Early single-stage systems exhibited either excessive false positives or near-zero verified yield, motivating the adoption of a two-stage verification pipeline. The shared-bias limitation of a homogeneous detector–detector cascade was addressed using a heterogeneous detection-to-classification architecture combining YOLOv12-large with EfficientNet-B2. On the respective evaluation datasets, this redesign increased first-stage detection mAP from 32.7% to 69.5%, reduced the second-stage false-positive rate from 99.96% to 32.26%, and decreased the manual inspection workload by approximately 70%. Redis-based asynchronous processing, pre-aggregated reporting, and migration to TensorFlow Lite further supported national-scale deployment. Future work should conduct statistically powered multi-route trials, distinguish model non-detection from data non-reception, and evaluate both stages on a common held-out dataset.

## Figures and Tables

**Table 1:** Deployed homogeneous cascade versus the proposed heterogeneous cascade.

Aspect	Deployed (Homogeneous)	Proposed (Heterogeneous)
First stage	YOLOv7-tiny (detection)	YOLOv12-large (detection)
Second stage	YOLOv7 (detection)	EfficientNet-B2 (classification)
Second-stage input	Detected image	Detected image + candidate region
Second-stage output	Bounding box [x, y, w, h]	Inherited bounding box + pothole probability and class label
Verification mechanism	Re-detection (shared bias)	Region classification (independent task)
Primary objective	Localization accuracy	Candidate detection and false-positive suppression

**Table 2:** Experiment 2 (2024): detected count, missed count, and system-level yield by vehicle type and mount. Encounters = potholes × 3 passes (pre-repair GT = 8 → 24; repaired = 22 → 66). Missed = encounters − detected (aggregates model non-detection and non-reception). Abbreviations: GT, ground truth.

Vehicle	Mount	Pre-Rep. Det.	Missed	Yield	Rep. Det.	Missed	Yield
Truck (Porter)	Improved	14	10	58.3%	13	53	19.7%
	Baseline	0	24	0.0%	5	61	7.6%
Van (Starex)	Improved	13	11	54.2%	20	46	30.3%
	Baseline	1	23	4.2%	5	61	7.6%
Passenger car (K3)	Improved	10	14	41.7%	17	49	25.8%
	Baseline	0	24	0.0%	0	66	0.0%

**Table 3:** Matched-ROI comparison of candidate verifier backbones (identical protocol; 15,112-crop validation set). Precision, recall, and F1 are for the pothole class; latency is per-image GPU inference. The deployed EfficientNet-B2 is in bold. Abbreviations: GPU, graphics processing unit; ROI, region of interest.

Backbone	Params (M)	Precision	Recall	F1	Macro-F1	Latency (ms)
MobileNetV3-Large	4.2	91.2	89.9	90.5	72.2	2.92
EfficientNet-B5	28.34	92.6	87.2	89.8	72.9	10.70
**EfficientNet-B2 (deployed)**	**7.7**	**92.7**	**86.1**	**89.3**	**72.3**	**6.99**
ResNet-50	23.51	93.3	83.8	88.3	71.7	2.88
EfficientNet-B4	17.55	94.4	80.2	86.7	70.7	8.89

**Table 4:** Component-level ablation on field data. Each row varies one component relative to the deployed homogeneous cascade; detector and verifier metrics lie on different tracks and are not summed. Abbreviations: mAP, mean average precision.

Change	Component Varied	Metric Affected	Before	After
(a) First-stage detector upgrade	YOLOv7-tiny → YOLOv12-large	Detection mAP/Recall/Precision	32.7%/47.7%/37.3%	69.5%/78.9%/61.6%
(b) Second-stage verifier: task change	YOLOv7 detector → EfficientNet-B2 classifier	Second-stage false-positive rate	99.96%	32.26% (−67.7%p)
(b′) Verifier classifier quality (standalone)	EfficientNet-B2 (classification)	Precision/Recall/F1-score	—	73.6%/74.3%/73.9%

**Table 5:** Candidate models evaluated for each stage and the rationale for the adopted choice. Abbreviations: FP, false positive; FPN, feature pyramid network; ONNX, Open Neural Network Exchange; SSD, single shot multibox detector; TFLite, TensorFlow Lite.

Stage	Candidate	Trade-Off	Decision
First stage (detector, edge)	YOLOv7-tiny	Fast but low recall on small/far potholes	Baseline (replaced)
	SSD/older YOLO	Comparable speed, weaker small-object recall	Rejected
	Faster R-CNN (two-stage)	Higher accuracy, latency incompatible with real-time edge	Rejected
	YOLOv12-large	Improved backbone/neck/FPN, better small-object recall, TFLite-exportable	Adopted
Second stage (verifier, server)	YOLOv7 (detector)	Shares first-stage bias; confirms rather than removes false positives	Baseline (replaced)
	MobileNet-class classifier	Low latency, weaker fine-grained texture discrimination	Rejected
	EfficientNet-B4/B5, large ResNet	More capacity, higher latency, no commensurate FP reduction	Rejected
	EfficientNet-B2 (classifier)	Different-task breaks shared bias; strong accuracy at deployable cost; ONNX export	Adopted

**Table 6:** Correspondence between reported results and their measurement populations.

Result	Population/Data	Size	Year	Ground Truth
Exp. 1 device yield (Table 8)	Common road route, transmitted candidates	32/370 candidates	2022	Visual + KICT verification
Exp. 2 system-level yield (Table 2 and Table 8)	Paju Dayul-dong route, 3 passes	8 + 22 potholes (24 + 66 enc.)	2024	Visual road inspection
First-stage detection (Table 10)	Curated detection test images	276 images	2025	Annotated boxes
Second-stage false-positive rate (Table 11)	Operational driving data	January–February 2025 (labeled evaluation-set size not specified in the available operational records; see Section 6)	2025	Verification labels
Deployed-cascade metrics (Table 12)	Curated evaluation images	100 images/config	2023	Annotated boxes

**Table 7:** Controlled held-out end-to-end evaluation on 2550 unseen frames (detector confidence 0.25; verifier threshold 0.5; matches at IoU ≥ 0.5). The heterogeneous verifier reduces false positives by 27.3% and raises precision while retaining 91.7% of recall; because it only removes candidates, it is a precision-oriented filter. Abbreviations: IoU, intersection over union.

Configuration	Precision	Recall	F1	False Positives
Detector only (YOLOv12-large)	0.709	0.663	0.685	2745
Cascade (+EfficientNet-B2 verifier)	0.755	0.608	0.673	1996

**Table 8:** Experiment 1 (2022): early single-stage devices versus visual ground truth on a common route.

Metric	Action-Cam + Laptop	Compact IoT (KICT Verified)
Candidates transmitted	32	370
Candidates verified as potholes	7	0
Unverified candidates	25	370
Verified yield	22.0%	0.0%

**Table 9:** Experiment 2 system-level yield with 95% percentile bootstrap confidence intervals (20,000 resamples). Zero-detection cells degenerate to [0.0, 0.0] under the percentile bootstrap and are shown as observed zeros. Pre-repair encounters = 24; repaired encounters = 66 per vehicle. Abbreviations: CI, confidence interval.

Vehicle	Mount	Pre-Repair Yield [95% CI]	Repaired Yield [95% CI]
Truck (Porter)	Improved	58.3% [37.5, 79.2]	19.7% [10.6, 30.3]
	Baseline	0.0% (obs. 0)	7.6% [1.5, 13.6]
Van (Starex)	Improved	54.2% [33.3, 75.0]	30.3% [19.7, 40.9]
	Baseline	4.2% [0.0, 12.5]	7.6% [1.5, 15.2]
Passenger car (K3)	Improved	41.7% [20.8, 62.5]	25.8% [15.2, 36.4]
	Baseline	0.0% (obs. 0)	0.0% (obs. 0)
Pooled (3 vehicles)	Improved	51.4% [40.3, 62.5]	—
	Baseline	1.4% [0.0, 4.2]	—

**Table 10:** First-stage (edge) detector before and after upgrade. Test set: 276 images; confidence threshold 0.1; IoU threshold 0.4. Figures are for the lightweight edge model and are not comparable to the earlier server-detector baseline.

Metric	YOLOv7-Tiny (Before)	YOLOv12-Large (After)	Change
mAP (detection)	32.7%	69.5%	+36.8%p
Recall	47.7%	78.9%	+31.2%p
Precision	37.3%	61.6%	+24.3%p

**Table 11:** Second stage before and after redesign, and standalone classifier metrics. False-positive rate measured on operational driving data collected in 2025.

Item	YOLOv7 Detector (Before)	EfficientNet-B2 Classifier (After)
Second-stage task	Object detection	Binary classification (pothole vs. background)
False-positive rate	99.96%	32.26% (−67.7%p)
Precision (classifier)	—	73.6%
Recall (classifier)	—	74.3%
F1 (classifier)	—	73.9%

**Table 12:** Deployed-cascade detection metrics (background; superseded by Section 5.4). Each configuration is evaluated on 100 images.

Configuration	Evaluation Images	Detections	mAP	Precision	Recall
2022 detection baseline	—	—	0.514	—	0.59 (F1-score)
2023—app (stage 1)	100	186	0.5743	0.6156	0.571
2023—second-stage filter (stage 2)	100	122	0.5975	0.5831	0.6094

## Data Availability

The data supporting the findings of this study are not publicly available because they contain operational road-management information. Additional information may be available from the corresponding author upon reasonable request, subject to institutional approval.

## Supplementary Materials

The following supporting information can be downloaded at: https://www.mdpi.com/article/10.3390/s26175573/s1. Supplementary Text S1: Model architectures; S2: Dataset composition; S3: Two-stage transfer-learning procedure (EfficientNet-B2); S4: Data augmentation; S5: Input resolution and ROI cropping; S6: Export, quantization, and deployment; S7: Numeric training hyperparameters not in the recovered materials (transparency note); S8: Matched-ROI backbone comparison, including Table S1 (matched-ROI comparison of candidate verifier backbones); S9: Development and server hardware and software.

## Abbreviations

The following abbreviations are used in this manuscript: YOLO, You Only Look Once; mAP, mean average precision; IoU, intersection over union; NMS, non-maximum suppression; FPN, feature pyramid network; ROI, region of interest; ONNX, Open Neural Network Exchange; TFLite/LiteRT, TensorFlow Lite; MMS, mobile mapping system; GIS, geographic information system; GNSS, global navigation satellite system; IMU, inertial measurement unit.
