# Supplementary material for: Vehicle-Mounted Vision Sensing for Large-Scale Road Surface Monitoring: A Multi-Year Field Study of Acquisition Geometry and a Heterogeneous Detection–Classification Pipeline for Pothole Mapping
Source: Sensors (Basel). 2026 Sep 2;26(17):5573. doi: 10.3390/s26175573 (PMC13567972; doi:10.3390/s26175573)
Supplement: Supplementary file 1 [file sensors-26-05573-s001.zip › sensors-4496069-supplementary.pdf]

Supplementary Materials

Manuscript ID: sensors-4496069

Title: Vehicle-Mounted Vision Sensing for Large-Scale Road Surface Monitoring: A Multi-Year Field Study of Acquisition Geometry and a Heterogeneous Detection–Classification Pipeline for Pothole Mapping

This document reports the training and deployment configuration of the two-stage pothole-monitoring pipeline, documented from the recovered project engineering materials (the region-of-interest datasets and the inference/deployment pipeline). It is provided in support of Reviewer 1 (Comments 2 and 4) and Reviewer 2 (Comment 1). Where a numeric setting is not part of the recovered materials, this is stated explicitly rather than estimated; no value below is inferred or reconstructed.

S1. Model architectures

Stage	Location	Model	Role
Stage 1	Vehicle edge device	YOLOv12-large	Permissive real-time pothole detector (high recall)
Stage 2	Server	EfficientNet-B2	Binary verifier (pothole vs. background) on cropped regions of interest (ROIs)

The first stage is tuned for high recall so that few true potholes are missed; the second stage, a task-heterogeneous classifier, suppresses the resulting false positives. The two stages are decoupled by an asynchronous message queue (see S6).

S2. Dataset composition

Stage 1 — detector (YOLOv12-large) training frames

Subset	Frames
Existing (reused)	2856
Newly collected	12,753

Subset	Frames
**Total**	**15,609**

Stage 2 — classifier (EfficientNet-B2) ROI crops

Class	Crops	Composition
Background	8564	Manholes, shadows, sealed cracks, tire marks
Pothole	49,467	Confirmed pothole ROIs
**Total (training)**	**58,031**	
Validation	15,112	Held-out ROI crops

The operational road imagery is restricted by institutional confidentiality policy and cannot be released; this table provides a documented dataset description in lieu of the raw imagery.

S3. Two-stage transfer-learning procedure (EfficientNet-B2)

The server verifier was trained by transfer learning in two stages (main manuscript, Section 3.3):

1. **Feature-extractor stage** — the ImageNet-pretrained backbone is frozen and only the classification head is trained on the ROI crops.
2. **Fine-tuning stage** — the full network is unfrozen and fine-tuned end-to-end on the ROI crops.

The exact numeric optimization settings for these stages are addressed in S7.

S4. Data augmentation

The augmentation pipeline documented in the project records (main manuscript, Section 4.4) comprised standard geometric and photometric transforms — horizontal flipping and intensity normalization — extended with blur, rotation, and low-light (reduced-brightness) transforms intended to reflect operational acquisition variability (motion blur, viewing-angle variation, and low-illumination driving). The precise per-transform probabilities and parameter ranges are not part of the recovered materials (which comprise the datasets and the inference/deployment code rather than the original training scripts).

S5. Input resolution and ROI cropping

- **Classifier input resolution:** 224 × 224 (ROI crop).
- **ROI cropping workflow:** the first-stage detector bounding box defines the region that is cropped and resized to 224 × 224 for the second-stage classifier (main manuscript, Figure 4).
- The recovered deployment pipeline applies **no additional contextual padding**: the first-stage detector bounding box is cropped directly and resized to 224 × 224 with ImageNet normalization before classification.

S6. Export, quantization, and deployment

Component	Framework path	Deployment target
Edge detector (YOLOv12-large)	TorchScript → TensorFlow Lite (quantized)	Vehicle-mounted edge device
Server verifier (EfficientNet-B2)	PyTorch → ONNX	Server

The two stages are decoupled by an asynchronous Redis message queue (main manuscript, Section 3.4): the edge device transmits detected ROIs to the server, which performs verification independently of capture. Confirmed observations are matched to the responsible road-management office by a PostGIS spatial join and stored in a PostgreSQL/PostGIS management database (main manuscript, Section 3.1).

S7. Numeric training hyperparameters not in the recovered materials (transparency note)

The exact numeric hyperparameters of the original 2025 production training — optimizer, learning-rate schedule, batch size, and epoch count for both YOLOv12-large training and EfficientNet-B2 fine-tuning — are **not part of the recovered materials**, which comprise the training datasets and the inference/deployment code rather than the original training scripts. These items are noted as a reproducibility limitation in the main manuscript (Section 6). Every configuration detail that *is* available is reported in Sections S1–S6 above, and the protocol under which the matched-ROI backbone comparison (S8) was produced is reported there; no numeric value is estimated in their place.

S8. Matched-ROI backbone comparison

Following recovery of the project's region-of-interest dataset and deployment pipeline, a controlled matched-ROI comparison of five candidate verifier backbones was carried out under a single identical protocol: input 224 × 224 with ImageNet normalization (matching the deployed verifier), ImageNet-pretrained initialization with full-network fine-tuning, class-weighted cross-entropy for the 5.8:1 background-to-pothole imbalance, AdamW at a learning rate of $1{\times}10^{-4}$, batch size 128, and six epochs, evaluated on the 15,112-crop held-out validation set. Precision, recall, and F1 below are for the pothole class; latency is per-image GPU inference on the comparison hardware. These are comparison-hardware GPU latencies only; they are not field or on-device latencies and are not comparable to the deployment latency discussed in the main manuscript. This comparison appears in the main manuscript as Table 2.

Table S1. Matched-ROI comparison of candidate verifier backbones.

Backbone	Params (M)	Precision	Recall	F1	Macro-F1	GPU latency (ms)
MobileNetV3-Large	4.2	91.2	89.9	90.5	72.2	2.92
EfficientNet-B5	28.34	92.6	87.2	89.8	72.9	10.70
**EfficientNet-B2 (deployed)**	**7.7**	**92.7**	**86.1**	**89.3**	**72.3**	**6.99**
ResNet-50	23.51	93.3	83.8	88.3	71.7	2.88
EfficientNet-B4	17.55	94.4	80.2	86.7	70.7	8.89

The candidate backbones perform comparably (pothole-class F1 between 86.7% and 90.5%); the deployed EfficientNet-B2 sits at a strong accuracy-per-parameter operating point, and the larger ResNet-50 and EfficientNet-B4/B5 yield no F1 improvement despite two to four times the parameters. For comparison, the standalone verifier metrics on the project's own operational data were EfficientNet-B2 precision 73.6%, recall 74.3%, F1 73.9% (main manuscript, Table 11), together with the second-stage false-positive-rate reduction from 99.96% to 32.26%.

S9. Development and server hardware and software

Item	Specification
CPU	Intel Core i9-10900X
Memory	64 GB RAM
Storage	1 TB SSD

Item	Specification
Server / inference GPU	NVIDIA GeForce RTX 3060 (12 GB)
CUDA	10.1
PyTorch	1.7.1
TensorFlow	2.2.0

The specific vehicle-mounted edge device (make, model, and system-on-chip) is **not documented in the recovered materials** and is therefore left unspecified rather than estimated (main manuscript, Sections 4.6 and 6).
